# The Optimal Approach for Laparoscopic Adrenalectomy through Mono Port regarding Left or Right Sides: A Comparative Study

**DOI:** 10.1155/2014/747361

**Published:** 2014-08-20

**Authors:** Wooseok Byon, Keehoon Hyun, Ji-Sup Yun, Yong Lai Park, Chan Heun Park

**Affiliations:** Department of Surgery & Breast-Thyroid Cancer Center, Kangbuk Samsung Hospital, Sungkyunkwan University School of Medicine, 108 Pyung-Dong, Jongno-Gu, Seoul 110-746, Republic of Korea

## Abstract

*Introduction.* Several studies have shown the feasibility and safety of both transperitoneal and posterior retroperitoneal approaches for single incision laparoscopic adrenalectomy, but none have compared the outcomes according to the left- or right-sided location of the adrenal glands.* Materials and Methods.* From 2009 to 2013, 89 patients who received LAMP (laparoscopic adrenalectomy through mono port) were analyzed. The surgical outcomes attained using the transperitoneal approach (TPA) and posterior retroperitoneal approach (PRA) were analyzed and compared.* Results and Discussion.* On the right side, no significant differences were found between the LAMP-TPA and LAMP-PRA groups in terms of patient characteristics and clinicopathological data. However, outcomes differed in which LAMP-PRA group had a statistically significant shorter mean operative time (84.13 ± 41.47 min versus 116.84 ± 33.17 min; *P* = 0.038), time of first oral intake (1.00 ± 0.00 days versus 1.21 ± 0.42 days; *P* = 0.042), and length of hospitalization (2.17 ± 0.389 days versus 3.68 ± 1.38 days; *P* ≤ 0.001), whereas in left-sided adrenalectomies LAMP-TPA had a statistically significant shorter mean operative time (83.85 ± 27.72 min versus 110.95 ± 29.31 min; *P* = 0.002).* Conclusions.* We report that LAMP-PRA is more appropriate for right-sided laparoscopic adrenalectomies due to anatomical characteristics and better surgical outcomes. For left-sided laparoscopic adrenalectomies, however, we propose LAMP-TPA as a more suitable method.

## 1. Introduction

Since the report of the first pioneering experiences [1], laparoscopic adrenalectomy has become the gold standard for the treatment of benign adrenal tumors. Owing to further development in surgical techniques and instruments, as well as a general increase of interest toward minimally invasive procedures in the past decades, a single incision approach was developed [2] and has been applied to conventional laparoscopic adrenalectomy.

A single incision approach has many advantages compared to conventional procedures, including improved cosmesis, reduced postoperative pain, and quicker convalescence [3]. However, single incision adrenalectomy necessitates more advanced techniques compared with multiport laparoscopic surgery [4, 5]; therefore careful selection of an adequate surgical approach is the key to a successful operation.

The aim of this study was to describe surgical techniques, to analyze the outcomes, and to provide insight on the optimal choice of surgical approach for each individual patient receiving laparoscopic adrenalectomy through mono port (LAMP).

## 2. Materials and Methods

### 2.1. Patients

From March 2009 to November 2013, 107 patients underwent laparoscopic adrenalectomy in our institute. Among them, 18 patients received conventional laparoscopic adrenalectomy, respectively. The remaining 89 patients, who received laparoscopic adrenalectomy through mono port (LAMP), were included in our study. All operations were performed by a single surgeon.

All patients received LAMP with either the transperitoneal approach or the posterior retroperitoneal approach, regardless of side. Initially, the surgical approach was chosen according to the tumor size and body habitus of the patient. For patients with tumors larger than 4 cm, BMI over 30 kg/m^2^, or with thick subcutaneous back tissue (thickness over 4 cm between the skin and Gerota's fascia), the transperitoneal approach was indicated. We selected patients with tumors less than 4 cm, BMI under 30 kg/m^2^, or with previous abdominal operations for the posterior retroperitoneal approach. However, after allocating patients to each approach according to the selection criteria for 33 cases, due to the small number of patients with tumors larger than 4 cm (*N* = 7), patients with BMI over 30 kg/m^2^ (*N* = 1), or patients with abundant retroperitoneal fat (*N* = 1), an imbalance occurred between groups, after which patients were randomly selected to receive each surgical approach, except for patients with tumors over 7 cm (*N* = 3), who received LAMP using the transperitoneal approach.

The surgical outcomes attained using the transperitoneal and posterior retroperitoneal approaches according to the right and left sides were analyzed and compared with respect to tumor size, operation time, time to first oral intake, postoperative hospitalization time, estimated blood loss, and postoperative complications. Statistical analysis was performed using SPSS ver. 18.0, and *P* values of <0.05 were considered statistically significant.

### 2.2. Operation Methods

#### 2.2.1. Laparoscopic Adrenalectomy through Mono Port Using the Transperitoneal Approach (LAMP-TPA)

Under general anesthesia, the patient was positioned in the lateral (right or left) decubitus position. The operation table was flexed just above the level of the iliac crest to maximally widen the space between the iliac crest and the costal margin for the best possible port access. After padding all pressure points (shoulders, elbows, hips, ankles, etc.) to prevent nerve injury and pressure sores, a bean bag placed under the patient before surgery was made firm to securely position the patient on the operation table.

**Figure 1:**
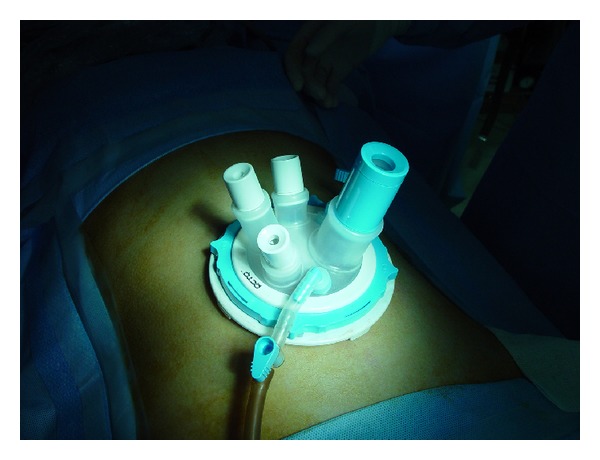
OCTO port.

Using preoperative abdominal CT scans, we measured the length from the adrenal tumor to the umbilicus, and with this measurement as a landmark, the selection of the optimal incision location was made. Usually, a single 3 cm incision is made parallel to the lower costal margin, two fingerbreadths below the costal margin in the midclavicular line. After opening the peritoneum, an OCTO port (Dalim Surgnet, Korea) (Figure 1) was inserted into the access site, and pneumoperitoneum to 15 mmHg was established using carbon dioxide insufflation.

For the left adrenal gland, the splenic flexure of the colon and the spleen were mobilized and drawn anteromedially to expose the retroperitoneum. For the right-sided adrenalectomy, after the right lobe of the liver was mobilized, it was retracted superiomedially with a snake retractor to expose the adrenal gland and the inferior vena cava. After exposing the adrenal gland, the feeding vessels were ligated using a Ligasure (Covidien, Mansfield, MA, USA). The central vein of the adrenal gland was usually ligated with conventional laparoscopic clips. After adequate hemostasis, the specimen was retrieved using a retrieval pouch.

#### 2.2.2. Laparoscopic Adrenalectomy through Mono Port Using the Posterior Retroperitoneal Approach (LAMP-PRA)

Under general anesthesia, the patient was placed prone in the jackknife position, and the operation table was flexed to maximize exposure of the posterior retroperitoneal space from the subcostal margin to the iliac crest. A 3 cm sized transverse skin incision was made one fingerbreadth below the lowest tip of the 12th rib. The fascia was incised, and the external oblique, internal oblique, and transversalis muscles were bluntly divided to expose the retroperitoneal space. After minimal working space in the retroperitoneum was made for port insertion, the OCTO port was inserted and CO_2_ was insufflated to 15 mmHg for pneumoretroperitoneum. After creating adequate working space, Gerota's fascia was opened, and the kidney upper pole was mobilized to expose the adrenal gland. After dissecting the adrenal gland from the surrounding tissue, the adrenal central vein was identified and ligated. Finally, the adrenal gland was placed in a pouch and retrieved.

## 3. Results

From March 2009 to November 2013, 89 patients underwent LAMP. Forty-seven patients received surgery for left-sided adrenal tumors and 42 patients for right-sided tumors. In cases of left-sided adrenalectomy, the transperitoneal and posterior retroperitoneal approaches were performed in 26 and 21 patients, and on the left, 19 and 23 patients received surgery using each approach, respectively.  Table 1 shows the clinical and pathological patient characteristics of all patient groups.

In cases of left-sided adrenalectomy, there were no statistical differences regarding patient factors in both groups. In pathologic diagnosis, one patient in the LAMP-TPA group was diagnosed with metastatic adenocarcinoma and one patient in the LAMP-PRA group with metastatic hepatocellular carcinoma.

With regard to adrenalectomies performed on the right side, no significant differences were found between the two groups except for patient age (TPA 40.79 ± 5.53 years versus PRA 50.22 ± 11.61 years; *P* = 0.002).

The mean tumor sizes of the left-sided groups were similar (3.28 ± 1.72 cm versus 3.45 ± 2.34 cm; *P* = 0.779) (Table 2). However, the LAMP-TPA group had a shorter mean operative time than the LAMP-PRA group, which was shown to be statistically significant (83.85 ± 27.72 min versus 110.95 ± 29.31 min; *P* = 0.002). In contrast, the average time to first oral intake was shorter in the LAMP-PRA group (1.10 ± 0.30 days versus 1.81 ± 0.49 days; *P* ≤ 0.001), but there were no differences in hospitalization time (3.62 ± 1.02 days versus 3.57 ± 0.75 days; *P* = 0.870) and estimated blood loss (26.15 ± 13.21 mL versus 25.72 ± 12.53; *P* = 0.743) between both groups.

In right-sided adrenalectomies, the mean tumor sizes were 2.72 ± 1.57 cm in the LAMP-TPA group and 2.61 ± 1.19 cm in the LAMP-PRA group (*P* = 0.811), respectively (Table 2). The LAMP-PRA group had a significantly shorter mean operative time (84.13 ± 41.47 min versus 116.84 ± 33.17 min; *P* = 0.008). Also, the average time to first oral intake (1.00 ± 0.00 days versus 1.21 ± 0.42 days; *P* = 0.042) and hospitalization time (2.17 ± 0.39 days versus 3.68 ± 1.38 days; *P* ≤ 0.001) were shorter in the LAMP-PRA group. Both groups had similar estimated blood losses (23.28 ± 11.74 mL versus 24.52 ± 7.48 mL; *P* = 0.240).

Regarding complications, one patient in the left LAMP-TPA suffered from postoperative ileus and required longer hospitalization, but no additional postoperative complications were reported in the other groups.

## 4. Discussion

In the search for better patient outcomes, surgery for benign adrenal diseases has gradually changed from the conventional open method to the less invasive methods of laparoscopic adrenalectomy. The laparoscopic approach offers several advantages over open adrenalectomy, such as reduced blood loss, fewer complications, less postoperative pain, and a shorter period of hospital stay [6–8].

To further enhance these advantages, less invasive methods have been introduced, leading to the development of the single incision laparoscopic adrenalectomy [2].

Several studies have shown the feasibility and safety of both transperitoneal and posterior retroperitoneal approaches in single incision laparoscopic adrenalectomy [4, 9–13], but none have compared the outcomes according to the left or right side. Our results show that there may be a difference in surgical results depending on the approach selected according to which side the surgery is performed. We propose that the reason for this disparity is due to the anatomical location and characteristics of the adrenal glands.

The left kidney is located approximately between the vertebral level T12 to L3, and due to the asymmetry within the abdominal cavity caused by the liver, the right kidney is slightly lower than the left [14]. Also, the left kidney is typically slightly larger than the right [15]. Both adrenal glands are located superior to the kidneys. The right adrenal gland is pyramidal in shape and lies at the apex of the right kidney. The left adrenal gland has a semilunar shape and lies on the superomedial or anterosuperomedial aspect of the left kidney. On the right side, the liver is located in front of the right adrenal gland, making the anterior margin, which is just separated by the parietal peritoneum. On the left, the spleen with the pancreatic tail makes up the anterior border to the left adrenal gland.

**Figure 2:**
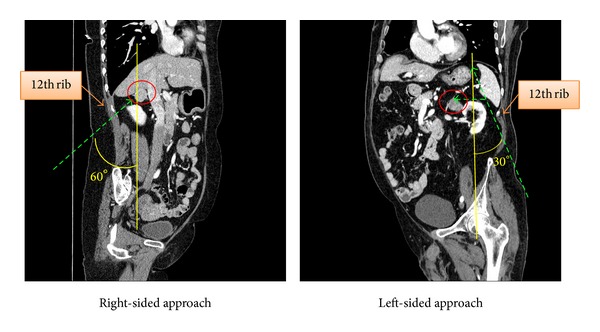
Comparison of right and left-sided LAMP-PRA.

Due to this anatomical presentation, in the posterior retroperitoneal approach, the right adrenal gland is closer with respect to the subcostal margin, and because of its location just superior to the apex of the right kidney, visualization of the adrenal gland is more feasible on the right compared to the left (Figure 2). Also, by using this approach, the right adrenal gland can be directly accessed without mobilization of any intra-abdominal viscera, typically the liver, which can be time consuming and prone to complications.

According to our data, on the right side, the PRA showed statistical significant improvement in operation time compared to the TPA and also demonstrated better postoperative results.

As for the left adrenal gland, however, the retroperitoneal approach can be somewhat challenging. In single incision laparoscopic adrenalectomy, the incision site is limited to the inferior border of the thoracic cage, and it cannot be made directly above the adrenal glands. Thus, in general, the location of the gland is relatively more further from the incision site on the left side. Also, the field of vision and working space are usually narrow [13], and the range of movement is limited by the incision site itself. Due to this difference in surgical depth, a more acute angle is needed to manipulate the gland on the left (Figure 2). Furthermore, direct visualization of the adrenal gland is usually obscured by the apex of the left kidney, so access can only be achieved by excessive retraction of the left kidney during surgery. On the other hand, the TPA, due to the relative familiarity of surgical anatomy to most surgeons, provides a more feasible access to the adrenal gland, even though it requires dissection of the spleen, pancreas tail and splenic flexure of the colon.

According to our data, the operative time was significantly shorter in the LAMP-TPA group on the left side. Postoperative outcomes were similar, although, the time to first oral intake was shorter in the LAMP-PRA group. This was probably due to CO_2_ gas insufflation and manipulation of intra-abdominal organs (colon, spleen, pancreas, etc.) which resulted in delayed peristalsis.

**Figure 3:**
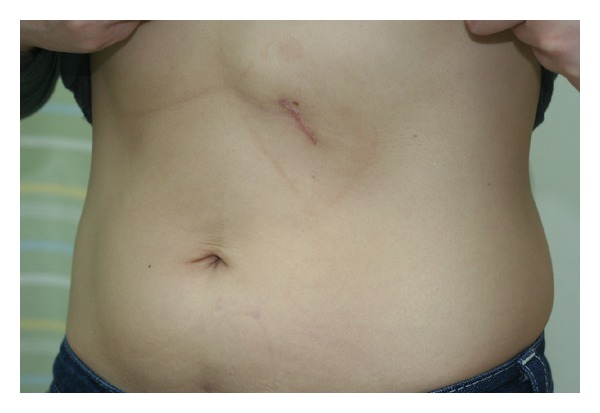
Postoperative scar of left-sided LAMP-TPA.

There have been some negative views regarding the need for a single incision approach in laparoscopic adrenalectomy, since the conventional approach is feasible and has good outcomes [6–8]. But, in order to evacuate the specimen after conventional laparoscopic adrenalectomy, an additional incision or elongation of the previous incision is needed, which could result in longer operation times and disfigurement of the postoperative wound. After initial trocar insertion in LAMP, however, no further disruption of the wound is needed, resulting in excellent cosmesis (Figure 3). Furthermore, direct visualization of the abdominal layers during opening of the wound as well as identification of the underlying structures during placement of the trocar is possible, which could reduce trocar-related complications. As seen in this study, no wound related complications were reported in all groups, which supports this finding.

## 5. Conclusion

In conclusion, we report that the LAMP-PRA is a more adequate approach for right-sided single incision laparoscopic adrenalectomies. Although the anterior approach may offer greater familiarity of the anatomy to the surgeon, visibility of the right adrenal gland is solely achieved through retraction of the liver, which can be avoided with a posterior approach.

For left-sided adrenalectomies, however, we propose that the LAMP-TPA is more suitable. The anatomical location of the left adrenal gland hinders feasible access through the posterior approach. In contrast, the transperitoneal approach provides easier manipulation of the adrenal gland, which could result in better results for the patient.

## Figures and Tables

**Table 1:** Patient characteristics.

	Left (*N* = 47)		*P* value	Right (*N* = 42)		*P* value
	LAMP-TPA	LAMP-PRA		LAMP-TPA	LAMP-PRA	
Patients (*N*)	26	21		19	23	
Sex (*N*)			0.326			0.327
Male	11	9		15	15	
Female	15	12		4	8	
Age (years)	48.35 ± 12.26	53.38 ± 11.55	0.158	40.79 ± 5.53	50.22 ± 11.61	0.002
BMI (kg/m^2^)	23.95 ± 3.35	27.28 ± 8.20	0.093	25.93 ± 2.71	25.31 ± 2.99	0.489
Diagnosis			0.936			0.509
Primary aldosteronism	6	3		3	5	
Cushing's syndrome	12	10		4	8	
Pheochromocytoma	3	4		0	0	
Nonfunctioning tumor	4	3		12	10	
Others	1	1		0	0	

LAMP-TPA: laparoscopic adrenalectomy through mono port using the transperitoneal approach, LAMP-PRA: laparoscopic adrenalectomy through mono port using the posterior retroperitoneal approach.

**Table 2:** Outcomes after surgery.

	Left (*N* = 47)		*P* value	Right (*N* = 42)		*P* value
	LAMP-TPA (*N* = 26)	LAMP-PRA (*N* = 21)		LAMP-TPA (*N* = 19)	LAMP-PRA (*N* = 23)	
Tumor size (cm)	3.28 ± 1.72	3.45 ± 2.34	0.779	2.72 ± 1.57	2.61 ± 1.19	0.811
Operative time (min)	83.85 ± 27.72	110.95 ± 29.31	0.002	116.84 ± 33.17	84.13 ± 41.47	0.008
Time to first oral intake (days)	1.81 ± 0.49	1.10 ± 0.30	<0.001	1.21 ± 0.42	1.00 ± 0.00	0.042
Length of hospitalization (days)	3.62 ± 1.02	3.57 ± 0.75	0.870	3.68 ± 1.38	2.17 ± 0.39	<0.001
Estimated blood loss (mL)	26.15 ± 13.21	25.72 ± 12.53	0.743	24.52 ± 7.48	23.28 ± 11.74	0.240
Complications	1	0		0	0	

LAMP-TPA: laparoscopic adrenalectomy through mono port using the transperitoneal approach, LAMP-PRA: laparoscopic adrenalectomy through mono port using the posterior retroperitoneal approach.
